# Number sense: the mediating effect between nonverbal intelligence and children’s mathematical performance

**DOI:** 10.1186/s41155-022-00231-1

**Published:** 2022-09-14

**Authors:** Hui Zhou, Qiutong Tan, Xiaolin Ye, Lujia Miao

**Affiliations:** 1 Center for Brain, Mind and Education, Shaoxing University, Shaoxing City, 312000 People’s Republic of China; 2 Department of Psychology, School of Teacher Education, Shaoxing University, Shaoxing City, 312000 People’s Republic of China; 3 School of Teacher Education, Huzhou University, Huzhou City, 313000 People’s Republic of China; 4 Research Center of Education Evaluation and Rural Education Development, Zhejiang Agriculture and Forestry University, Hangzhou City, 311300 People’s Republic of China

**Keywords:** Number sense, Nonverbal intelligence, Mathematical performance, Mediating effect, Primary school

## Abstract

The study explored the mediating effect of number sense between nonverbal intelligence and children’s mathematical performance. The sample consisted of 131 pupils in Shaoxing City of China from grades 1, 3, and 5. The students completed measures of nonverbal intelligence, number sense, basic arithmetic ability, mathematical performance, rapid automatized naming, and working memory. Results show that although all variables significantly relate with each other (all *p* < .01), only nonverbal intelligence, number sense, and basic arithmetic ability significantly affect children’s mathematical performance (all *p* < .01). According to multiple-mediation model, nonverbal intelligence significantly predicts children’s mathematical performance through number sense and basic arithmetic ability. These findings suggest that domain-specific mathematical skills play a prominent role in children’s mathematical performance in primary school, rather than domain-general cognitive functions. Educators should pay attention to develop children’s number sense in order to improve children’s mathematical ability.

## Introduction

Solving mathematical problems plays an important role in children’s learning and living. Previous studies have found that fluid intelligence such as nonverbal intelligence is the core trait of individual differences, which significantly predict children’s mathematics performance in primary school (Lechner et al., 2019; Primi et al., 2010). However, the influence mechanism of nonverbal intelligence on children’s mathematical ability is still unclear. In addition to nonverbal intelligence, number sense is also an important factor affecting children’s mathematical performance. Number sense is affected by congenital factors and also promoted through education. From birth, children have a primitive and prelinguistic capacity about understanding and representing numbers (Clarke & Beck, 2021). After practice, children become greater precision in number representation. Better number sense supports children’s arithmetic ability well and further could promote performance in more complex mathematical problem. Previous studies proposed number sense, and cognitive ability has different effects on mathematics in preschool children and adults (Manginas et al., 2021; van Bueren et al., 2022). It suggests number sense may have phased characteristics and different effects on children’s mathematics in primary school. Therefore, we aim to explore the effect of number sense between the relationship of nonverbal intelligence and children’s mathematical performance in primary school children.

### The relationship between nonverbal intelligence and children’s mathematical performance

Intelligence refers to a general mental ability, which is important in individual practical affairs of life (Simpson-Kent et al., 2020). Compared with verbal intelligence which involves language ability, nonverbal intelligence mainly processes visual information or spatial information (Blazhenkova & Kozhevnikov, 2010). Intelligence could be a causal factor in children’s learning performance (Watkins et al., 2007). When facing novel and complex tasks, intelligence has a high correlation with learning achievement (Snow et al., 1984), especially mathematics. Several studies suggest that intelligence, especially nonverbal intelligence, is an important predictor of math achievement (Lechner et al., 2019; Peng et al., 2019). For example, a longitudinal study employed differential reasoning test and found children with higher intelligence score increase faster in math achievement than children with lower intelligence score over 2 years (Primi et al., 2010). Through a longitudinal study of individuals aged 6–21, compared with age, vocabulary, and spatial skills, results found that intelligence is the only predictor of future mathematical achievement, and the influence range of intelligence spans primary and secondary schools (Green et al., 2017). However, the internal mechanism of how nonverbal intelligence influence on children’s mathematics is still unclear.

Nonverbal intelligence plays a substantial role in individual differences. Researches on intelligence uncovered biological correlates between genetics and intelligence. For example, according to study of children’s intelligence heritability from age 5 to 18, Hoekstra et al. (2007) suggest that genetic influence could completely attribute to the stability of intelligence. Studies of intelligence development indicate that genetic influences are unique to IQ rather than environmental influences such as socioeconomic status (Deary et al., 2009; Van der Sluis et al., 2008).

Children’s ability to solve complex mathematical problems requires formal education and experience. For example, in primary school, there are phasic changes in children’s mathematics learning. In the first stage, children begin to learn basic numerical symbols and concepts. In the second stage, children try to use mathematical principles and strategies. By the third stage, based on several mathematical knowledge and experience, children start to solve complex mathematical problems (Reys et al., 2014). The influence of nonverbal intelligence on children’s mathematical performance may be indirect. For example, researchers propose that intelligence is associated with initial learning level but not with the growth rate (Tamez et al., 2008). According to twin samples study, Lukowski et al. (2017) proposed number sense has moderate genetic trait and accounts for children’s mathematics and cognitive ability by genetic mechanism. However, number sense has unique effect with mathematics beyond intelligence. It suggests number sense could be a mediatorial role between nonverbal intelligence and mathematical performance. In addition to genetic influence, number sense has a special effect on mathematical performance. Therefore, the present study focuses on number sense influence mechanism between nonverbal intelligence and mathematical performance.

### Number sense

Number sense refers to the ability to quickly understand, estimate, and manipulate numerical values (Dehaene, 2001). Number sense contains multiple components. Berch (2005) proposed there are high-order and low-order divisions of number sense based on previous researches of mathematical cognition, cognitive development, and mathematical education. Low-order number sense is based on biological sensory organs. It is an approximate number system shared by humans and animals (Feigenson et al., 2004). For example, even children without formal math education can choose more chocolate chips from two biscuits or choose more biscuits box in boxes with different biscuits numbers. Besides human beings, the same phenomenon has been found in animals. So far, amphibians, birds, reptiles, and mammals have shown number sense ability (Nieder, 2021). Through the sense of number, animals can obtain higher survival opportunities and reproduction possibilities in finding food, avoiding predation, hunting prey, and social interaction (Nieder, 2020). Human beings also take the system as the basic core of numerical knowledge, which makes it possible to obtain a higher level of mathematical ability later (Wong et al., 2017).

The high-order number sense refers to the mathematical concepts meaning construction, which is acquired by learning and understanding of mathematical principles, numerical relations, mathematical laws, and so on (Berch, 2005). Although there is a common number sense mechanism between humans and animals, only humans learn to understand and master more complex mathematical skills with age. Researchers view number sense as a skill rather than an “intrinsic” process (Robinson et al., 2002). Along with children’s development, number sense will be affected by various social factors such as educational strategy and cultural environment (Jordan et al., 2012; Aunio et al., 2006). Therefore, children’s number sense will reflect in the fluency and flexibility of numerical operation and calculation process, as well as the final mathematical performance.

It can be seen that children’s number sense not only has congenital physiological origin but also is affected by acquired factors such as educational factor. Therefore, the present study focuses on how children become skilled in mathematics and the role of number sense in this process.

### Children’s basic arithmetic ability

Children’s basic arithmetic ability is a special mathematical skill, which refers to computing an addition or subtraction number fact in limit time (e.g., 8 + 7, 15–7) (Cowan et al., 2011; Sorvo et al., 2017). In order to get answers, children retrieve the number facts from memory or calculate them through simple strategies, such as 5 + 5 = 10 so 5 + 6 = 11 (Baroody, 1999; Clarke et al., 2016; Levine et al., 1992). In primary school, there is a close relationship between basic arithmetic ability and children’s mathematics performance. Deficits in mastery of arithmetic facts are key characteristic of children with mathematics problem (Gilmore et al., 2010).

The number sense view of Baroody (2006) proposes that number sense is the basis of children’s basic arithmetic ability. Before formal education, children’s number sense of quantity is relevant to their arithmetic ability. For example, they can distinguish which set contains more elements than the other set (Sprenger & Benz, 2020; Brannon et al., 2004). However, basic arithmetic skill requires the transformation of sets by adding or subtracting elements (Levine et al., 1992). Based on the number sense view, mathematics education enables children to further learn to calculate and manipulate number on the basis of number representation. For example, if children have difficulties in manipulating nonverbal number representation, they could be limit in calculation fast (Cohen et al., 2000). Nonverbal intelligence is also an important factor affecting children’s basic arithmetic ability. A study of the third-grade children in primary school found that nonverbal intelligence test score of children with higher arithmetic task score was significantly higher than that of children with lower arithmetic task score (Jordan et al., 2003).

To sum up, basic arithmetic ability is the basis of children’s mathematical ability. It could develop from number sense and closely relate with nonverbal intelligence. However, there is still uncertain role of basic arithmetic ability in the relationship between nonverbal intelligence, number sense, and mathematics performance. Therefore, the current research assumes that number sense could affect children’s mathematical performance through basic arithmetic ability.

### Related cognitive ability with mathematics

Children’s mathematical performance is affected not only by nonverbal intelligence but also domain-specific mathematical skills and other cognitive functions (Geer et al., 2019; Hawes et al., 2015; Green et al., 2017).

Rapid automatized naming and working memory are important influence factor of children’s mathematics. They are core factors in cognitive development and affect children’s learning with growth (Tourva & Spanoudis, 2020; Tamez et al., 2008).

Rapid automatized naming (RAN) refers to the ability to name familiar visual stimuli such as numbers, colors, objects, and letters as quickly as possible (e.g., Cui et al., 2017; Liao et al., 2015). RAN requires the ability to learn arbitrary associations between a visual stimulus and a spoken response. The fast visual-verbal association is base on quickly retrieve stimulus labels fluently from memory (e.g., a number’s verbal label). Several studies found rapid automatized naming continues to predict mathematical achievement, especially children’s basic arithmetic ability, even after controlling working memory, executive functions, reading, and so on (Cui et al., 2017; van der Sluis et al., 2004). For example, Malone et al. (2021) investigated the relationship between RAN and mathematical ability of primary school children in grade 3. They propose that RAN is closely related to children’s numerical ordering ability and affects the relationship of numerical ordering ability and arithmetic. A meta-analysis from 38 studies shows a significant correlation between RAN and mathematics and suggests RAN as an early predictor of mathematical skill (Koponen et al., 2017).

Working memory defined as a mental workspace for individuals to control, regulate, or maintain relevant information in complex tasks accomplishment (Raghubar et al., 2010). Working memory has close relationship with intelligence (Tourva & Spanoudis, 2020) and is a strong predictor of mathematical skills in primary school period (Friso-Van den Bos et al., 2013). A Longitudinal study of Chilean Children compare mathematics difficult children with typical developed children from the first grade (Guzmán et al., 2019). The result found working memory and RAN have significant contributions to differentiating two group mathematics performance. Therefore, when exploring the mediating effect of number sense on the relationship between nonverbal intelligence and mathematical performance of primary school children, the present study also tests children’s RAN and working memory to avoid possible confusion.

### Goal of the study

We aim to explore the mediating effect of number sense on the relationship between nonverbal intelligence and mathematical performance of primary school children. Our hypotheses are as follows:

1. Number sense has a significant mediating effect on the relationship between nonverbal intelligence and mathematical performance of primary school children.
2. Number sense affects children’s mathematical performance need through basic arithmetic ability.
3. Although working memory and rapid automatized naming have significant relationship of children’s mathematical ability, they cannot affect the mediating effect of number sense between non-verbal intelligence, and children’s mathematical performance.

## Method

### Participants

The current study randomly selects134 pupils in Shaoxing City of China. Two children were excluded for missing data more than two tests, and one child was excluded for intelligence score 5% lower than peers. Finally, the data of 131 pupils participated in the data analysis. These pupils are primary school students in the first, third, and fifth grade, including 44 in the first grade (53% girls; mean age of 7.2 years), 42 in the third grade (45% girls; mean age of 9.4 years), and 45 in the fifth grade (47% girls; mean age of 11.3 years). According to the teacher’s report, the subjects had normal or corrected visual acuity and had no physical or mental illness. Both children and their parents agreed to take tests and signed informed consent.

### Measurement

#### Nonverbal intelligence

Children’s nonverbal intelligence was tested by Raven progressive matrix test (combined) (Raven & Court, 1938). During the test, children need to observe the album of each patterns and select which small pictures can fill in patterns’ gaps. The test has no time limit, and the score is the total correct number (alpha coefficient, *α* = 0.96).

#### Number sense

Children were test with the number sets test (Geary et al., 2009). The test includes target numbers and object sets. The object set is composed of Arabic numerals and different number objects (circles, diamonds, triangles, and stars). Children need to find the correct number of object sets matching the target number as soon as possible and circle it with a pen. Before the formal test, children will perform two exercises of target numbers 4 and 3 to ensure their understanding of the test requirement. The test records children’s completing time and accuracy. In order to avoid ceiling effect of test accuracy, children’s completing time is employed to reflect individual differences (alpha coefficient, *α* = 0.95).

#### The basic arithmetic ability

The basic arithmetic task (Aunola & Räsänen, 2007) is designed for primary school children. Children only have 3 min time to complete addition and subtraction problems (a total of 50 items). The task score is the total correct item number completed by children. Previous studies on primary school children show that the task has a higher test-retest reliability than 0.94 (Aunola et al., 2004). In current study, alpha coefficient is 0.98 of the test.

#### Mathematical performance

Children’s mathematical performance was tested by the numerical operations subtest of the Wechsler Individual Achievement Test, Second Edition (WIAT-II; Wechsler, 2005). Numerical operations subtest is a well indicator of quantitative knowledge and mathematical performance (Parkin & Beaujean, 2012). In the test, children need to solve several problems of calculation and simple equations involving basic operations such as addition, subtraction, multiplication, and division. The test score is the total correct answer number (alpha coefficient, *α* = 0.98).

#### Working memory

The digit span test from the WISC-IV (Wechsler, 2004) is administered to children. The digit span test includes two tasks (digit forward recall task and digit backward recall task). Children is required to repeat digits in the same order after experimenter reading in digit forward recall task and repeat in backward order in digit backward recall task. The test records the correct number of items (alpha coefficient, *α* = 0.76).

#### RAN

The rapid automatized naming (RAN) task is administered to assess children’s processing speed (Denckla & Rudel, 1976). The child is presented with pages of numbers, colors, or words. They need to read stimulus quickly and correctly. The task records completed time and accuracy of children (alpha coefficient, *α* = 0.87).

### Procedure and data analysis

The primary school children in grades 1, 3, and 5 are randomly selected. Tests are conducted one on one between children and experimenters in a quiet room. Before each test, experimenters will introduce the test and how to complete it. Only after completing some exercises to ensure children’s understanding the formal test can be carried out. Experimenters are college students who received special training in advance. IBM SPSS Statistics for Windows version 22.0 was used to analyze the data. The analysis methods include descriptive statistical analysis, correlation analysis, and regression analysis. Furthermore, the PROCESS of SPSS (Hayes, 2017) was used for mediation model analysis.

## Results

The current study analyzed the data in three stages. First, we analyzed the variables’ correlation to confirm the relationships between nonverbal intelligence, number sense, basic arithmetic ability, and mathematical performance. Second, we conducted regression analyses to predict mathematical performance from nonverbal intelligence, number sense, basic arithmetic ability, and control variables. Last, we tested whether number sense mediated the association between nonverbal intelligence and children’s mathematical abilities.

### Correlation analysis of children’s number sense, mathematical ability, and other variables

As Table 1 indicates, current study analyzes the descriptive statistics and correlation of children’s nonverbal intelligence, number sense, basic arithmetic ability, mathematical performance, working memory, and processing speed. Correlation results show that the total time of children to complete number sense test is significantly negative which correlates with the accuracy of nonverbal intelligence test, basic arithmetic test, and mathematical test. Children need more completing time; they perform worse in number sense test. The result suggests children perform worse in number sense; they could also get fewer correct scores in nonverbal intelligence test, basic arithmetic test, and mathematical test. Correlation results also show that the accuracy score of children’s working memory test and RAN time are significantly correlated with accuracy scores of children’s nonverbal intelligence test, basic arithmetic test, and mathematical test. It suggests that children’s cognitive ability is significantly correlated with children’s mathematical abilities.

**Table 1:** Correlation analysis of variables

	*M*	*SD*	1	2	3	4	5	6
1NI	34.076	9.981	1	−0.550**	0.554**	0.687**	0.406**	−0.578**
2NS	397.134	201.596	−0.550**	1	−0.629**	−0.706**	−0.319**	0.635**
3BA	36.076	24.334	0.554**	−0.629**	1	0.805**	0.340**	−0.530**
4MP	15.038	6.390	0.687**	−0.706**	0.805**	1	0.353**	−0.613**
5WM	3.894	1.598	0.406**	−0.319**	0.340**	0.353**	1	−0.374**
6RAN	81.135	22.177	−0.578**	0.635**	−0.530**	−0.613**	−0.374**	1

*Note*: *NI* nonverbal intelligence, *NS* number sense, *BA* basic arithmetic ability, *MP* mathematical performance, *WM* working memory, *RAN* rapid automatized naming; *M* mean, *SD* standard deviation; *p < .05, **p < .01

### Regression analyses of mathematical performance on nonverbal intelligence, number sense, and basic arithmetic ability

The present study further analyzes the regression effect of nonverbal intelligence, number sense, and basic arithmetic ability in children’s mathematical performance. As Table 2 shows, even control variables of working memory and RAN, scores of nonverbal intelligence, number sense, and basic arithmetic affect children’s mathematical performance significantly. The result suggests that comparing cognitive abilities, mathematical domain-specific skills have a significant impact on pupils’ mathematical performance.

**Table 2:** Regression analyses of mathematical performance on nonverbal intelligence, number sense, and basic arithmetic ability

Model	B	SD	Beta	*t*	*p*
(Constant)	9.190	2.382		3.858**	.000
NI	0.167	.037	0.262	4.473**	.000
NS	−.007	.002	−0.207	−3.272**	.001
BA	0.131	.016	0.498	8.410**	.000
WM	−.068	0.193	−.017	−0.351	0.726
RAN	−.021	.017	−.074	−1.216	0.226

*Note*: *NI* nonverbal intelligence, *NS* number sense, *BA* basic arithmetic ability, *WM* working memory, *RAN* rapid automatized naming; *p < .05, **p < .01

### The mediating effect of number sense between nonverbal intelligence and children’s mathematical performance

Based on previous analyses, number sense and basic arithmetic ability are tested as potential mediators of the association between nonverbal intelligence and mathematical performance. The analysis used 5000 resamples to estimate 95% confidence intervals. If zero is not present in the 95% confidence intervals, the indirect effect is significantly different from zero at *p* < 0.05 (Preacher & Hayes, 2008). According the present multiple-mediation model, without intermediary variables, nonverbal intelligence has a significant direct predictive effect on children’s mathematical performance (path coefficient 0.687, *p* < 0.01). When the mediating variable of number sense enters in the model, nonverbal intelligence predicts number sense significantly (path coefficient −0.548, *p* < 0.01), and number sense predicts mathematical performance significantly (path coefficient −0.236, *p* < 0.01). The result suggest that for more score in nonverbal intelligence test, children need less time to complete the number sense test and could got more score in mathematical test. When the mediating variable of basic arithmetic ability enters in the model, nonverbal intelligence could predict basic arithmetic ability significantly (path coefficient 0.298, *p* < 0.01), and basic arithmetic ability significantly predicts mathematical performance (path coefficient 0.505, *p* < 0.01). The result suggests more scores in nonverbal intelligence test, and children perform better in the basic arithmetic test and could get more scores in mathematical test. When both mediating variables enter in the model, nonverbal intelligence predicts number sense significantly (path coefficient −0.548, *p* < 0.01), number sense predicts basic arithmetic ability significantly (path coefficient −0.467, *p* < 0.01), and basic arithmetic ability predicts mathematical performance significantly (path coefficient 0.505, *p* < 0.01). The result shows that nonverbal intelligence affects children’s mathematical performance development through number sense and basic arithmetic ability.

**Fig. 1:**
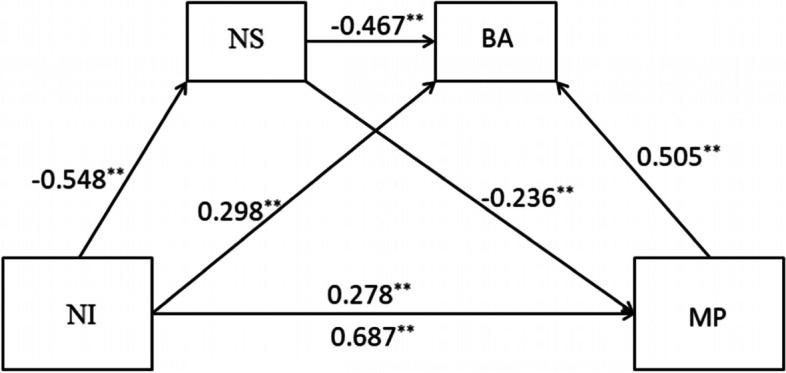
The chain multiple mediation model. Note: NI, nonverbal intelligence; NS, number sense; BA, basic arithmetic ability; MP, mathematical performance; *p < .05, **p < .01

As Table 3 shows, there are three indirect effects: nonverbal intelligence→number sense→mathematical performance (mediating effect accounted for 31.54% total effect), nonverbal intelligence→basic arithmetic ability→mathematical performance (mediating effect accounted for 36.67% total effect), and nonverbal intelligence→number sense→basic arithmetic ability→mathematical performance (mediating effect accounted for 31.54% total effect) Fig. 1.

**Table 3:** The mediating effect of number sense and basic calculation between nonverbal intelligence and mathematical performance

Effect	Path	Effect value	Boot SE	Boot LLCI	Boot ULCI	Relative effect
Total effect		0.409	0.040	0.338	0.496	
Indirect effect	NI→NS→MP	0.129	0.064	0.071	0.293	31.54%
Indirect effect	NI→BA→MP	0.150	0.062	0.030	0.252	36.67%
Indirect effect	NI→NS→BA→MP	0.129	0.026	0.077	0.178	31.54%

Note: *NI* nonverbal intelligence, *NS* number sense, *BA* basic arithmetic ability, *MP* mathematical performance

## Discussion

The current study examines the associations between nonverbal intelligence, number sense, basic arithmetic ability, and mathematical performance. Results show that children’s nonverbal intelligence, number sense, basic arithmetic ability, and mathematical score are significantly correlated with each other. Even control working memory and RAN, scores of nonverbal intelligence, number sense, and basic arithmetic affect children’s mathematical performance significantly. In multiple-mediation model, nonverbal intelligence significantly predicts children’s mathematical performance through number sense and basic arithmetic ability.

The main finding of this study is the mediating effect of number sense and basic arithmetic ability on the association between nonverbal intelligence and mathematical performance. The result is consistent with previous studies. Firstly, nonverbal intelligence is significantly associated with children’s academic performance. However, the influence of nonverbal intelligence on children’s academic achievement may not be direct. For example, a study of 836 Chinese students show that intelligence predicted children’s academic performance in Chinese, Math, and English, and the personality has an interaction effects with intelligence (Zhang & Ziegler, 2015). In addition to the stable characteristics of individual differences such as nonverbal intelligence, various skills children learn and master have a more direct impact on their final academic performances. For example, a meta-analysis of children’s intelligence relation with reading and mathematics is found, although intelligence showed a stronger relation to mathematics than to reading, education, and learning experience will have a greater impact on children’s academic achievement with growth (Peng et al., 2019).

Second, number sense has congenital physiological characteristics; it also teaches learning mathematical skills such as basic arithmetic skills. Children have innate understanding of number magnitude and representation (Buijsman, 2021; Clarke & Beck, 2021; Malone et al., 2020). Through repeated mapping of symbolic number with nonsymbolic magnitude, such as Arabic number 5 with five apples, children can learn to operate arithmetic questions (Malone et al., 2019). Proficiency in nonsymbolic to symbolic mapping could further predict children’s mathematics performance (Mazzocco et al., 2011). The current results suggest that number sense might play a role early on children acquiring mathematical ability.

Finally, nonverbal intelligence affects children’s mathematical ability through mathematical skills in special fields, rather than general cognitive function such as working memory and process speed. Although intelligence such as reasoning has close relationship with working memory and process speed (Conway et al., 2003; Schubert et al., 2017), reasoning, especially nonverbal spatial reasoning, plays an important role in children’s development and learning (Demetriou et al., 2022). For example, a study of early school education found children’s reasoning ability significantly relates with computational thinking and arithmetic fluency (Xu et al., 2022). Studies found that there is a close relationship between cognitive function and children’s mathematical ability, especially in the preschool stage (Blankson & Blair, 2016; Manginas et al., 2021). Consistent with previous studies, the current study found that both domain-general cognitive function and domain-specific mathematical skills are closely related to children’s mathematical ability. However, our results show that only number sense has a mediating effect between nonverbal intelligence and children’s mathematical performance. The possible explanation is that number sense plays a prominent role in children’s mathematical performance at a specific stage, that is, in primary school. For example, van Bueren et al. (2022) found number sense, and working memory has no mediating effect in mathematical ability of older children and adults.

### Limitations and future recommendations

The present study has several limitations should be taken into account. Firstly, the proposed causal relationship between nonverbal intelligence, number sense, basic arithmetic ability, and mathematics performance needs longitudinal studies to prove evident. The present cross-sectional study cannot directly observe the development of children’s mathematical ability in the whole primary school stage. Secondly, the current study only controls working memory and rapid automatized naming; there are other cognitive factors that could affect the relationship such as attention. Future researches need to determine which other cognitive variables could be important. Finally, the present study employed typical tests to examine children’s abilities, such as the digit span test to examine children’s working memory. However, working memory includes multiple sub-components, such as the spatial working memory. Raven’s test examines children’s spatial and configurational intelligence, which may have functional overlap with the spatial working memory. Future research can employ a variety of tests to examine children’s single ability, so as to avoid the impact of research tools on results.

## Conclusion

In sum, the current study reveals mediating effect of number sense between nonverbal intelligence, basic arithmetic ability, and mathematics performance. Compared to domain-general cognitive functions, domain-specific basic mathematical skills play a prominent role in children’s mathematical performance in primary school. These findings suggest that educators should pay attention to children’s development of number sense and basic arithmetic ability.

## Data Availability

The data and material that support the findings of this study are available from the corresponding author HZ, upon reasonable request.

## Abbreviation

RAN: Rapid automatized naming

## References

[CR1] Aunio P, Niemivirta M, Hautamäki J, Van Luit JE, Shi J, Zhang M (2006). Young children’s number sense in China and Finland. Scandinavian Journal of Educational Research.

[CR2] Aunola K, Leskinen E, Lerkkanen MK, Nurmi JE (2004). Developmental dynamics of math performance from preschool to grade 2. Journal of Educational Psychology.

[CR3] Aunola K, Räsänen P (2007). *The Basic Arithmetic Test*.

[CR4] Baroody AJ (1999). Children’s relational knowledge of addition and subtraction. Cognition and Instruction.

[CR5] Baroody AJ (2006). Why children have difficulties mastering the basic number combinations and how to help them. Teaching Children Mathematics.

[CR6] Berch DB (2005). Making sense of number sense: Implications for children with mathematical disabilities. Journal of Learning Disabilities.

[CR7] Blankson AN, Blair C (2016). Cognition and classroom quality as predictors of math achievement in the kindergarten year. Learning and Instruction.

[CR8] Blazhenkova O, Kozhevnikov M (2010). Visual-object ability: A new dimension of non-verbal intelligence. Cognition.

[CR9] Brannon EM, Abbott S, Lutz DJ (2004). Number bias for the discrimination of large visual sets in infancy. Cognition.

[CR10] Buijsman S (2021). The representations of the approximate number system. Philosophical Psychology.

[CR11] Clarke B, Nelson N, Shanley L (2016). Mathematics fluency—More than the weekly timed test. *The Fluency Construct*.

[CR12] Clarke S, Beck J (2021). The number sense represents (rational) numbers. Behavioral and Brain Sciences.

[CR13] Cohen L, Dehaene S, Chochon F, Lehericy S, Naccache L (2000). Language and calculation within the parietal lobe: A combined cognitive, anatomical and fMRI study. Neuropsychologia.

[CR14] Conway AR, Kane MJ, Engle RW (2003). Working memory capacity and its relation to general intelligence. Trends in Cognitive Sciences.

[CR15] Cowan R, Donlan C, Shepherd DL, Cole-Fletcher R, Saxton M, Hurry J (2011). Basic calculation proficiency and mathematics achievement in elementary school children. Journal of Educational Psychology.

[CR16] Cui J, Georgiou GK, Zhang Y, Li Y, Shu H, Zhou X (2017). Examining the relationship between rapid automatized naming and arithmetic fluency in Chinese kindergarten children. Journal of Experimental Child Psychology.

[CR17] Deary IJ, Johnson W, Houlihan LM (2009). Genetic foundations of human intelligence. Human Genetics.

[CR18] Dehaene S (2001). Précis of the number sense. Mind & Language.

[CR19] Demetriou A, Mougi A, Spanoudis G, Makris N (2022). Changing developmental priorities between executive functions, working memory, and reasoning in the formation of g from 6 to 12 years. Intelligence.

[CR20] Denckla MB, Rudel RG (1976). Rapid ‘automatized’ naming (RAN): Dyslexia differentiated from other learning disabilities. Neuropsychologia.

[CR21] Feigenson L, Dehaene S, Spelke E (2004). Core systems of number. Trends in Cognitive Sciences.

[CR22] Friso-Van den Bos I, Van der Ven SH, Kroesbergen EH, Van Luit JE (2013). Working memory and mathematics in primary school children: A meta-analysis. Educational Research Review.

[CR23] Geary DC, Bailey DH, Hoard MK (2009). Predicting mathematical achievement and mathematical learning disability with a simple screening tool: The number sets test. Journal of Psycho educational Assessment.

[CR24] Geer EA, Quinn JM, Ganley CM (2019). Relations between spatial skills and math performance in elementary school children: A longitudinal investigation. Developmental Psychology.

[CR25] Gilmore CK, McCarthy SE, Spelke ES (2010). Non-symbolic arithmetic abilities and mathematics achievement in the first year of formal schooling. Cognition.

[CR26] Green CT, Bunge SA, Chiongbian VB, Barrow M, Ferrer E (2017). Fluid reasoning predicts future mathematical performance among children and adolescents. Journal of Experimental Child Psychology.

[CR27] Guzmán B, Rodríguez C, Sepúlveda F, Ferreira RA (2019). Number sense abilities, working memory and RAN: A longitudinal approximation of typical and atypical development in Chilean children. Revista de Psicodidáctica (English ed.).

[CR28] Hawes Z, Moss J, Caswell B, Poliszczuk D (2015). Effects of mental rotation training on children’s spatial and mathematics performance: A randomized controlled study. Trends in Neuroscience and Education.

[CR29] Hayes AF (2017). *Introduction to mediation, moderation, and conditional process analysis: A regression-based approach*.

[CR30] Hoekstra RA, Bartels M, Boomsma DI (2007). Longitudinal genetic study of verbal and nonverbal IQ from early childhood to young adulthood. Learning and Individual Differences.

[CR31] Jordan NC, Glutting J, Dyson N, Hassinger-Das B, Irwin C (2012). Building kindergartners’ number sense: A randomized controlled study. Journal of Educational Psychology.

[CR32] Jordan NC, Hanich LB, Kaplan D (2003). Arithmetic fact mastery in young children: A longitudinal investigation. Journal of Experimental Child Psychology.

[CR33] Koponen T, Georgiou G, Salmi P, Leskinen M, Aro M (2017). A meta-analysis of the relation between RAN and mathematics. Journal of Educational Psychology.

[CR34] Lechner CM, Miyamoto A, Knopf T (2019). Should students be smart, curious, or both? Fluid intelligence, openness, and interest co-shape the acquisition of reading and math competence. Intelligence.

[CR35] Levine SC, Jordan NC, Huttenlocher J (1992). Development of calculation abilities in young children. Journal of Experimental Child Psychology.

[CR36] Liao CH, Deng C, Hamilton J, Lee CSC, Wei W, Georgiou GK (2015). The role of rapid naming in reading development and dyslexia in Chinese. Journal of Experimental Child Psychology.

[CR37] Lukowski SL, Rosenberg-Lee M, Thompson LA, Hart SA, Willcutt EG, Olson RK (2017). Approximate number sense shares etiological overlap with mathematics and general cognitive ability. Intelligence.

[CR38] Malone SA, Burgoyne K, Hulme C (2020). Number knowledge and the approximate number system are two critical foundations for early arithmetic development. Journal of Educational Psychology.

[CR39] Malone SA, Heron-Delaney M, Burgoyne K, Hulme C (2019). Learning correspondences between magnitudes, symbols and words: Evidence for a triple code model of arithmetic development. Cognition.

[CR40] Malone SA, Pritchard VE, Hulme C (2021). Separable effects of the approximate number system, symbolic number knowledge, and number ordering ability on early arithmetic development. Journal of Experimental Child Psychology.

[CR41] Manginas G, Papageorgiou A, Theodorou M, Iakovaki M (2021). Mathematical competence in preschool students and its relationship with intelligence, age and cognitive functions of attention, information processing speed and reaction inhibition.

[CR42] Mazzocco MM, Feigenson L, Halberda J (2011). Preschoolers’ precision of the approximate number system predicts later school mathematics performance. PLoS One.

[CR43] Nieder A (2020). The adaptive value of numerical competence. Trends in Ecology & Evolution.

[CR44] Nieder A (2021). Neuroethology of number sense across the animal kingdom. Journal of Experimental Biology.

[CR45] Parkin JR, Beaujean AA (2012). The effects of Wechsler Intelligence Scale for Children—Fourth Edition cognitive abilities on math achievement. Journal of School Psychology.

[CR46] Peng P, Wang T, Wang C, Lin X (2019). A meta-analysis on the relation between fluid intelligence and reading/mathematics: Effects of tasks, age, and social economics status. Psychological Bulletin.

[CR47] Preacher KJ, Hayes AF, Hayes AF, Slater MD, Synder LB (2008). Assessing mediation incommunication research. *The Sage sourcebook of advanced data analysis methods for communication research*.

[CR48] Primi R, Ferrão ME, Almeida LS (2010). Fluid intelligence as a predictor of learning: A longitudinal multilevel approach applied to math. Learning and Individual Differences.

[CR49] Raghubar KP, Barnes MA, Hecht SA (2010). Working memory and mathematics: A review of developmental, individual difference, and cognitive approaches. Learning and Individual Differences.

[CR50] Raven JC, Court JH (1938). *Raven's progressive matrices*.

[CR51] Reys R, Lindquist M, Lambdin DV, Smith NL (2014). *Helping Children Learn Mathematics*.

[CR52] Robinson CS, Menchetti BM, Torgesen JK (2002). Toward a two-factor theory of one type of mathematics disabilities. Learning Disabilities Research & Practice.

[CR53] Schubert A-L, Hagemann D, Frischkorn GT (2017). Is general intelligence little more than the speed of higher-order processing?. Journal of Experimental Psychology: General.

[CR54] Simpson-Kent IL, Fuhrmann D, Bathelt J, Achterberg J, Borgeest GS, Kievit RA (2020). Neurocognitive reorganization between crystallized intelligence, fluid intelligence and white matter microstructure in two age-heterogeneous developmental cohorts. Developmental Cognitive Neuroscience.

[CR55] Snow RE, Kyllonen PC, Marshalek B (1984). The topography of ability and learning correlations. Advances in the Psychology of Human Intelligence.

[CR56] Sorvo R, Koponen T, Viholainen H, Aro T, Räikkönen E, Peura P (2017). Math anxiety and its relationship with basic arithmetic skills among primary school children. British Journal of Educational Psychology.

[CR57] Sprenger P, Benz C (2020). Children’s perception of structures when determining cardinality of sets—Results of an eye-tracking study with 5-year-old children. ZDM.

[CR58] Tamez E, Myerson J, Hale S (2008). Learning, working memory, and intelligence revisited. Behavioural Processes.

[CR59] Tourva A, Spanoudis G (2020). Speed of processing, control of processing, working memory and crystallized and fluid intelligence: Evidence for a developmental cascade. Intelligence.

[CR60] van Bueren, N. E., van der Ven, S. H., Roelofs, K., Kadosh, R. C., & Kroesbergen, E. H. (2022). Predicting math ability using working memory, number sense, and neurophysiology in children and adults. *bioRxiv*. 10.1101/2022.02.10.479865. 10.3390/brainsci12050550 PMC9139259 35624937

[CR61] Van der Sluis S, De Jong PF, Van der Leij A (2004). Inhibition and shifting in children with learning deficits in arithmetic and reading. Journal of Experimental Child Psychology.

[CR62] Van der Sluis S, Willemsen G, De Geus EJ, Boomsma DI, Posthuma D (2008). Gene-environment interaction in adults’ IQ scores: Measures of past and present environment. Behavior Genetics.

[CR63] Watkins MW, Lei PW, Canivez GL (2007). Psychometric intelligence and achievement: A cross-lagged panel analysis. Intelligence.

[CR64] Wechsler D (2004). *Wechsler Scale of Intelligence*.

[CR65] Wechsler D (2005). *Wechsler Individual Achievement Test*.

[CR66] Wong TTY, Ho CSH, Tang J (2017). Defective number sense or impaired access? Differential impairments in different subgroups of children with mathematics difficulties. Journal of Learning Disabilities.

[CR67] Xu W, Geng F, Wang L (2022). Relations of computational thinking to reasoning ability and creative thinking in young children: Mediating role of arithmetic fluency. Thinking Skills and Creativity.

[CR68] Zhang J, Ziegler M (2015). Interaction effects between openness and fluid intelligence predicting scholastic performance. Journal of Intelligence.

